# Silencing and Innate Immunity in Plant Defense Against Viral and Non-Viral Pathogens

**DOI:** 10.3390/v4112578

**Published:** 2012-10-29

**Authors:** Anna S. Zvereva, Mikhail M. Pooggin

**Affiliations:** Institute of Botany, University of Basel, Schönbeinstrasse 6, 4056 Basel, Switzerland; Email: Anna.Zvereva@unibas.ch

**Keywords:** silencing, innate immunity, pattern-triggered immunity, effector-triggered immunity, siRNA, miRNA, plant antiviral defense, Cauliflower mosaic virus, silencing suppressor, avirulence protein

## Abstract

The frontline of plant defense against non-viral pathogens such as bacteria, fungi and oomycetes is provided by transmembrane pattern recognition receptors that detect conserved pathogen-associated molecular patterns (PAMPs), leading to pattern-triggered immunity (PTI). To counteract this innate defense, pathogens deploy effector proteins with a primary function to suppress PTI. In specific cases, plants have evolved intracellular resistance (R) proteins detecting isolate-specific pathogen effectors, leading to effector-triggered immunity (ETI), an amplified version of PTI, often associated with hypersensitive response (HR) and programmed cell death (PCD). In the case of plant viruses, no conserved PAMP was identified so far and the primary plant defense is thought to be based mainly on RNA silencing, an evolutionary conserved, sequence-specific mechanism that regulates gene expression and chromatin states and represses invasive nucleic acids such as transposons. Endogenous silencing pathways generate 21-24 nt small (s)RNAs, miRNAs and short interfering (si)RNAs, that repress genes post-transcriptionally and/or transcriptionally. Four distinct Dicer-like (DCL) proteins, which normally produce endogenous miRNAs and siRNAs, all contribute to the biogenesis of viral siRNAs in infected plants. Growing evidence indicates that RNA silencing also contributes to plant defense against non-viral pathogens. Conversely, PTI-based innate responses may contribute to antiviral defense. Intracellular R proteins of the same NB-LRR family are able to recognize both non-viral effectors and avirulence (Avr) proteins of RNA viruses, and, as a result, trigger HR and PCD in virus-resistant hosts. In some cases, viral Avr proteins also function as silencing suppressors. We hypothesize that RNA silencing and innate immunity (PTI and ETI) function in concert to fight plant viruses. Viruses counteract this dual defense by effectors that suppress both PTI-/ETI-based innate responses and RNA silencing to establish successful infection.

## 1. Pattern-Triggered Immunity (PTI)

Innate immunity is an evolutionary ancient mechanism that protects plants and animals from a wide range of pathogens. Invading pathogens are recognized by diverse pattern recognition receptors (PRRs) [1,2,3]. In plants, the first line of defense against non-viral pathogens relies on the recognition of conserved, pathogen-associated molecular patterns (PAMPs) by the transmembrane PRRs that include receptor-like kinases (RLKs) and receptor-like proteins [4,5]. For example, the bacterial flagellin-derived peptide flg22, one of the most studied PAMPs, is recognized by a complex composed of the RLK FLS2 (flagellin sensing 2) and the regulatory kinase BAK1 (BRI1-associated kinase 1). Specific binding of flg22 to FLS2 activates the FLS2-BAK1 complex eliciting PTI. Likewise, BAK1 is required for PTI upon specific recognition of the bacterial translation elongation factor Tu (EF-Tu)-derived peptide elf18 by the RLK EFR (elongation factor receptor). FLS2 and EFR belong to the superfamily of RLKs that contain the extracellular leucine-rich repeat (LRR) domain responsible for specific PAMP recognition and the intracellular kinase domain [2]. Within minutes of PAMP recognition, signaling cascades trigger a set of pathogen-related responses including elevation of intracellular Ca++ levels and production of reactive oxygen species (ROS), the activation of various kinases including CDKs (calcium-dependent) and MAPKs (mitogen-activated), the consecutive changes in phosphorylation states of many cellular proteins and broad changes in gene regulation, which leads to synthesis of various anti-microbial reagents [6,7,8]. 

In addition to PAMPs, the PTI system can recognize plant endogenous peptide (Pep) elicitors that are thought to amplify the defense responses against invading microorganisms [9]. In *Arabidopsis thaliana*, the Pep family members (AtPeps) are recognized by the RLKs PEPRs (Pep receptors) 1 and 2 which are structurally similar to FLS2 and EFR and also require BAK1 for transmitting Pep signals. Peps and other endogenous elicitors are collectively called danger-associated molecular patterns (DAMPs) [2]. It is conceivable that viral pathogens, for which no conserved PAMP was identified so far, may trigger PTI-based responses through activation of endogenous DAMPs.

## 2. Effector-Triggered Immunity (ETI)

To counteract PTI and establish robust infection in susceptible hosts, the pathogens deploy effector proteins (virulence factors) into the host cell. Some of the effector proteins block MAPK cascades by targeting RLKs and BAK1 [2]. In specific cases, plants have evolved resistance (R) genes that mediate intracellular recognition of effector proteins, which results in effector triggered immunity (ETI). ETI is a rapid and high-amplitude output, considered to be an amplified version of PTI [3,10]. The ETI signaling cascades often lead to hypersensitive response (HR) and programmed cell death (PCD) that locally counteracts pathogen attack and progression [11]. 

Among other factors, resistance to different pathogen types involves a regulation of the balance between salicylic acid (SA)- and ethylene/jasmonic acid (ET/JA)-dependent defense mechanisms [12]. SA is important for triggering HR/PCD upon effector recognition, thus mediating resistance against biotrophic pathogens. ET and JA play a role in the control of PCD spreading and regulate resistance against necrotrophic pathogens. All the three hormones regulate distinct sets of pathogen-related genes and are involved in triggering systemic-acquired resistance (SAR) that induces defenses in distal non-infected tissues after activation of local resistance [13]. Interestingly, SAR can also be induced by PAMP recognition and by local virus infection.

Most plant R proteins belong to the nucleotide binding site-leucine rich repeat (NB-LRR) family. Plant NB-LRRs are classified into two main classes: CC-NB-LRRs with the N-terminal coiled-coil (CC) domain and TIR-NB-LRRs with the N-terminal Toll-interleukin-1 receptor (TIR) domain, which have specialized functions in immune responses [3]. To explain how a relatively small number of NB-LRRs (*ca.* 160 in *A. thaliana*) could recognize thousands of different pathogens, a guard hypothesis (GH) was proposed [14] stating that the recognition is indirect, although examples of direct protein-protein recognition are also known. The GH postulates that NB-LRRs monitor the molecular outcome of the effector virulence functions: the effectors target the host factors, thus generating “modified self” signals responsible for NB-LRR activation [15]. The GH predicts that (a) a single NB-LRR can recognize the action of multiple unrelated effectors, (b) multiple NB-LRRs can monitor the same host target of effector action, and (c) evolutionary distant pathogens can target core components of the host cellular machinery. All these predictions are supported by studied cases of ETI [15].

In further support of GH, experimentally established interaction networks of the *A. thaliana* proteins and the bacterial and oomycete effectors reveal that independently evolved effectors converge onto hubs in the immune system network [16]. By extrapolation, viral effectors may also target the same hubs of the network.

One of the immune system hubs is the EDS1 (enhanced disease susceptibility 1) protein controlling both PTI-based responses that inhibit growth of virulent pathogens and ETI-based HR/PCD against avirulent pathogens. EDS1 forms two types of complexes, one on the plasma membrane (likely involved in PTI) and another with two TIR-NB-LRRs that ‘guard’ EDS1, and all these complexes are targeted by bacterial effectors [17,18]. Interestingly, nuclear-cytoplasmic shuttling of the EDS1/NB-LRR complex is required for elicitation of PCD [18]. Nuclear-cytoplasmic dynamics plays an important role in most of innate immune responses [19,3]. 

EDS1 and SA act redundantly in immune responses [20], and CC-NB-LRRs, which do not require EDS1 to elicit ETI, can potentially guard the SA pathway components [3]. Interestingly, CC-NB-LRRs of the ADR1 (activated disease resistance) subclade play a helper role in ETI and function in PTI-based defense. Notably, ADRs are required for ROS production and SA accumulation upon inoculation with effector-deficient bacteria [21]. This implies unconventional, ROS-mediated activation of NB-LRRs which induces SA-dependent PTI- and ETI responses [3]. We consider ADRs and components of the SA pathway as potential targets of viral effectors. 

Plant R proteins functioning depends on the chaperon complex that includes SGT1 (Suppressor of G2 allele of skp1), HSP90 (Heat-shock protein 90) and RAR1 (Required for Mla12 resistance). This complex might facilitate a conformational change of R proteins inducing the immune signaling after recognition of pathogen effectors or modified host proteins targeted by pathogen effectors. Mutations of the genes encoding the chaperone components strongly affect stability of R proteins [13]. Interestingly, SGT1 and HSP90 are essential for the mammalian inflammasome activity, linking the innate immune responses of these distant organisms [22]. These conserved components of immune system might also be targeted by viral effectors.

## 3. A Zig-Zag Model for Evolution of the Plant Immune System

**Figure 1:**
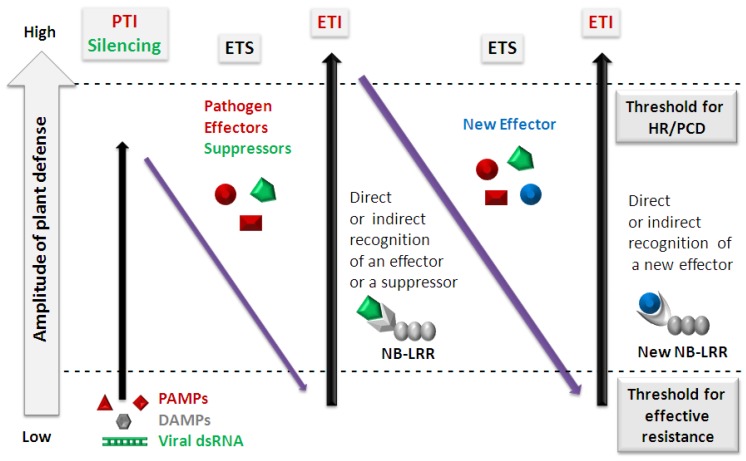
Zig-zag model for evolution of innate immunity- and silencing-based plant defense against viral and non-viral pathogens (adopted and extended from Jones and Dangl 2006 [10]). The ultimate amplitude of disease resistance or susceptibility is proportional to [PTI + Silencing – ETS + ETI]. In phase 1, plants detect pathogen-associated molecular patterns (PAMPs) and host danger-associated molecular patterns (DAMPs) via pattern-recognition receptors (PRRs) to induce pattern-triggered immunity (PTI) and, in the case of viral pathogens, plants additionally detect viral double-stranded RNA (dsRNA) to trigger RNA silencing. In phase 2, successful viral and non-viral pathogens deliver effectors/suppressors that interfere with both PTI and silencing, resulting in effector-triggered susceptibility (ETS). In phase 3, one effector or suppressor is recognized directly or indirectly by an NB-LRR protein, activating effector-triggered immunity (ETI), an amplified version of PTI that often passes a threshold for induction of hypersensitive response (HR) and programmed cell death (PCD). In phase 4, pathogen isolates are selected that have lost or modified the specifically recognized effector/suppressor, and perhaps gained a new effector that can help the pathogen to suppress ETI. A new plant NB-LRR allele is then evolved and selected that can recognize the newly acquired effector, resulting again in ETI.

The outcome of any plant-pathogen interaction depends on a relative contribution of susceptibility and resistance factors. In the zig-zag model, originally proposed by Jones and Dangl [10], PAMP (and DAMP) perception initiates the primary, PTI-based defense responses that limit (but do not fully stop) pathogen growth. Successful pathogens have evolved effector/virulence factors that promote pathogen growth by suppressing PTI, which results in effector-triggered susceptibility (ETS). To counteract the action of specific pathogen effectors plants have evolved ETI, a largely NB-LRR-based recognition of the ‘modified-self’ by-products of ETS. This evolutionary arms race between the host and the pathogen occurs in multiple rounds of ETS followed by ETI. The final outcome of the plant-pathogen interaction depends on the sum total of ([PTI − ETS] + ETI) [10,15]. We propose to extend this zig-zag model to plant-virus interactions (Figure 1).

## 4. Links between Plant Innate Immunity and RNA Silencing

RNA silencing is an evolutionary conserved, sequence-specific mechanism that regulates gene expression and chromatin states and defends against invasive nucleic acids such as transposons, transgenes and viruses [23,24,25]. Silencing is directed by 21-24 nt sRNAs processed from the double-stranded (ds)RNA precursors by Dicer or Dicer-like (DCL) enzymes. sRNAs associate with Argonaute (AGO) proteins and guide the resulting RNA-induced silencing complexes (RISC) to silence complementary RNA or DNA. In plants, silencing pathways generate two types of sRNAs: miRNAs and short interfering (si)RNAs. miRNAs are produced by DCL1 from hairpin dsRNA precursors transcribed by Pol II from *MIR* genes; they silence target genes through mRNA cleavage and/or translational repression. siRNAs of distinct size classes are processed by DCL4 (21-nt), DCL2 (22-nt) or DCL3 (24-nt) from dsRNA precursors produced by RNA-dependent RNA polymerases (RDRs) or from overlapping sense and antisense Pol II transcripts. RDR6-dependent, 21-nt trans-acting siRNAs (tasiRNAs) and secondary siRNAs silence genes post-transcriptionally (like miRNAs). RDR2-dependent, 24-nt heterochromatic siRNAs (hcsiRNAs) silence repetitive DNA transcriptionally through RNA-dependent DNA methylation (RdDM). RdDM involves plant-specific polymerases, Pol IV and Pol V, required for 24-nt siRNA production, amplification and action [26]. The *Arabidopsis* genome encodes 10 AGOs which are specialized in RdDM (AGO4/6/9), miRNA/tasiRNA pathways (AGO1/2/7) and other functions [27].

Growing evidence indicates that innate immunity and RNA silencing are closely linked. Various miRNAs and siRNAs have been implicated in innate immunity [28]. E.g., miR398 that targets superoxide dismutases is downregulated by ROS and plants overexpressing miR398 exhibit enhanced susceptibility to *Pseudomonas syringae * [29]. The bacterial flg22 induces miR393 that targets auxin receptors. The repression of auxin signaling activates the SA signaling that restricts *P. syringae* growth, which implicates auxin in disease susceptibility and miRNA-mediated suppression of auxin signaling in resistance [30]. Furthermore, miRNA-deficient *Arabidopsis* lines including *dcl1* restore growth of effector-deficient *P. syringae*, thus suggesting that DCL1 and miRNAs play a key role in PTI [31]. Notably, *P. syringae* effectors suppress biogenesis, stability, or activity of PAMP-responsive miRNAs [31]. Most miRNAs including miR393 and miR398 are bound to AGO1 and therefore AGO1 is a potential target of pathogen effectors. Interestingly, silencing suppressors of some RNA viruses target AGO1 (see below). Recently, AGO2-miR393* RISC was implicated in ETI response to avirulent *P. syringae* [32]. Likewise, AGO7 contributes to ETI, possibly through its function in the biogenesis of a long siRNA induced by avirulent *P. syringae* [28]. Since AGO1, AGO2 and AGO7 mediate antiviral defense (see below), it is conceivable that, in addition to their interaction with viral siRNAs, these AGOs contribute to anti-viral defense through endogenous miRNAs and siRNAs regulating PTI and ETI. 

RDR1 known to be involved in production of secondary viral siRNAs [33] is induced by SA, which implicates innate immunity signaling in silencing-based antiviral defense. Conversely, RDR6 mediates biogenesis of the endogenous siRNAs induced by bacterial effectors, which could account for enhanced growth of avirulent *P. syringae* on *Arabidopsis rdr6* mutant plants [28].

Emerging evidence implicates components of the nuclear silencing machinery in innate immunity. AGO4, in addition to its main function in RdDM, appears to have a distinct function in ETI in *N. benthamiana* [34]. Consistently, *Arabidopsis ago4* mutant plants exhibit reduced resistance to avirulent *P. syringae* and increase susceptibility to virulent *P. syringae*, whereas *rdr2* and *dcl3* mutations that abolish RdDM had no effect on bacterial growth [35]. Furthermore, *Arabidopsis* Pol V (but not Pol IV) contributes to JA-mediated resistance to fungi but counteracts SA-mediated resistance to virulent *P. syringae* [36].

Recent studies reveal that a large proportion of NB-LRR genes are silenced by miRNAs of the miR482 superfamily [37,38,39]. miRNA-directed silencing of NB-LRRs is associated with RDR6-dependent secondary siRNAs and can be partially suppressed by RNA viruses and virulent *P. syringae* [39]. Thus, plants seem to exploit the pathogen effector activities to achieve inducible expression of NB-LRRs. 

## 5. Plant Viruses and the PTI- and ETI-Based Defense System

In animals, the innate immune system is essential for initial detection of viruses and subsequent activation of adaptive immunity. Viral components, such as dsRNA, single-stranded RNA and DNA are sensed by three classes of receptors: retinoic acid-inducible gene I-like, Toll-like and nucleotide oligomerization domain-like receptors [40], with the latter being similar to plant NB-LRRs [3].In plants, there is no evidence for recognition of viral RNA or DNA by immune receptors and the RNA silencing system has evolved to recognize and target viral nucleic acids.

As noted above, no viral PAMP was identified so far and PTI-based antiviral responses can potentially be elicited by plant DAMPs. Additionally, ‘modified self’ products of the viral effector activity can be sensed by unconventional CC-NB-LRRs such as ADRs. Viral and non-viral pathogens induce similar immune reactions [41]. Thus, local virus infection leads to SAR in uninfected tissues. Furthermore, RNA viruses express Avr proteins that trigger HR/PCD or non-HR basal defense in resistance hosts. Like other pathogen effectors, viral Avr proteins are almost invariably virulence factors, which might suppress innate immune responses in susceptible hosts, and are recognized by NB-LRRs in resistant hosts. NB-LRRs confer resistance to highly divergent viruses through recognition of distinct viral proteins including coat, movement and replication proteins [41,42],which in some cases also act as suppressors of silencing (Table 1). 

For RNA viruses, the most studied *R* genes are tobacco *N* (TIR-NB-LRR), potato *Rx1* (CC-NB-LRR) and *Arabidopsis HRT* (CC-NB-LRR) which confer resistance to *Tobacco mosaic virus* (TMV), *Potato virus* X (PVX) and *Turnip crinkle virus* (TCV), respectively. The tobacco *N* mediates recognition of a helicase domain p50 of the TMV replicase p126/p180: interaction of the TMV effector with a chloroplastic protein leads to N protein activation and re-localization to the nucleus, which triggers PCD [43]. TMV p126 is a silencing suppressor and its helicase, methyltransferase and non-conserved domains appear to function redundantly in suppression [44]. In the case of PVX, the nuclear-cytoplasmic receptor protein Rx1 recognizes the viral coat protein (CP; not reported as silencing suppressor) [45,46]. Retention of Rx1 protein in the cytoplasm by Ran GTPase is required for ETI/PCD, suggesting that Ran is a ‘guardee’. In the case of TCV, the resistance protein HRT was proposed to guard a transcription factor, TCV-interacting protein (TIP): nuclear localization of TIP is inhibited by interaction with TCV CP, which leads to ETI [47]. However, a follow-up study has challenged these findings and, instead, proposed a role of TIP in basal (PTI-based) resistance to TCV and other RNA viruses [48]. TCV CP is also a silencing suppressor and its activities in ETI and silencing could be uncoupled [49]. TCV CP binding to AGO1, which would inactivate miRNA-AGO1 and viral siRNA-AGO1 RISCs, is required for TCV virulence [50]. Thus, TCV CP-mediated inactivation of AGO1-miR393 RISC may suppress antiviral PTI in susceptible hosts. 

**Table 1:** Cloned plant *R* genes and the viral protein effectors and silencing suppressors.

*R* gene	Plant	R protein structure	Virus, Family	Avr/Effector protein	Known silencing suppressor
*N*	*Nicotiana* sp.	TIR-NB-LRR	*Tobacco mosaic virus*, RNA tobamovirus	Replication protein (p126)	Replication protein (p126)
*Rx1, Rx2*	*Solanum tuberosum*	CC-NB-LRR	*Potato virus *X,RNA potexvirus	Coat protein	p25
*HRT*	*Arabidopsis* Dijon-17	CC-NB-LRR	*Turnip crinkle virus*,RNA carmovirus	Coat protein	Coat protein
*RCY1* *similar to HRT*	*Arabidopsis* C24	CC-NB-LRR	*Cucumber mosaic virus*, RNA cucumovirus	Coat protein	2b
*Sw-5*	*Solanum lycopersicum*	CC-NB-LRR	*Tomato spotted wilt virus*, RNA tospovirus	Replicase	NSs
*Y-1*	*Solanum tuberosum*	TIR-NB-LRR	*Potato virus* Y,RNA potyvirus	?	HC-Pro
*Tm-2*	*Solanum lycopersicum*	CC-NB-LRR	*Tomato mosaic virus*, RNA tobamovirus	Movement protein, Rep. (p126)	Rep. (p126)
*CYR1*	*Vigna mungo*	CC-NB-LRR	*Mungbean yellow mosaic virus*, DNA geminivirus	?	Transcriptional activator (AC2)
*?*	*Arabidopsis*	?	*Cauliflower mosaic virus*,DNA pararetrovirus	Transactivator/Viroplasmin, TAV	Transactivator/Viroplasmin, TAV

The potato *Y-1* gene (TIR-NB-LRR) confers resistance to the potyvirus *Potato virus* Y (PVY) [51], but an Avr protein of PVY or other RNA potyviruses is still unknown. *Arabidopsis* plants infected with the potyvirus *Turnip mosaic virus* (TuMV) could sustain growth of non-virulent *P. syringae*, which was interpreted as a negative effect of the TuMV-encoded silencing suppressor HCPro on the miRNA pathway [30]. Intriguingly, the anti-silencing activities of potyvirus HCPro and TCV CP require the ethylene-inducible transcription factor RAV2 and correlate with RAV2-dependent induction of defense genes [52]. HCPro-transgenic tobacco plants exhibit enhanced resistance to non-viral pathogens and *N*-mediated resistance to TMV, but, at elevated temperatures (inactivating *N*), enhanced susceptibility to TMV and other RNA viruses [53]. These findings suggest that HCPro may function as a PTI repressor in susceptible hosts and an Avr factor in resistant hosts. A recent study has revealed that HCPro interacts with the plant calmodulin-like protein rgs-CaM and this interaction leads to degradation of HCPro by autophagy, which interferes with HCPro silencing suppressor activity [54]. Furthermore, rgs-CaM interacts with other viral suppressors and is induced by infection with several RNA viruses [54]. Taken together, this host protein could be considered as a component of the innate immune system that recognizes viral effectors and triggers ETI-like responses. It is tempting to propose that rgs-CaM is guarded by an NB-LRR. 

Little is known about DNA viruses and plant innate immunity. In the case of geminiviruses (family *Geminiviridae*), the CC-NB-LRR gene *CYR1* was recently implicated in resistance to *Mungbean yellow mosaic India virus * [55]. To our knowledge no *R* gene was identified for pararetroviruses (family *Caulimoviridae*).

*Cauliflower mosaic virus* (CaMV) and *Rice tungro bacilliform virus *(RTBV) belong to distinct genera of *Caulimoviridae*, *Caulimovirus* and *Tungrovirus*, which differ in host range, insect vector and virion architecture [56]. Both viruses have 8 kb circular dsDNA genome that replicates via reverse transcription of pregenomic (pg)RNA. But they have different strategies of pgRNA translation and expression of distal ORF (ORF VI and ORF IV, respectively) [57,58]. The CaMV ORF VI product is a multifunctional protein that serves as a main component of viroplasm and a transactivator of pgRNA translation (hence named transactivator/viroplasmin, TAV). TAV is also a silencing suppressor [59,60,61] and an Avr protein. The TAV avirulence domain, which is also required for CaMV virulence and called ‘Avr/Vir’, was mapped within its variable N-terminus [62,63,64]. The following lines of indirect evidence suggest that TAV may suppress innate immune responses. It has been demonstrated that *A. thaliana *ecotype Tsu-0 is resistant to CaMV strain CM1841 but not W260 and that replacement of the N-terminal (Avr/Vir) domain of CM1841 TAV with the corresponding domain from W260 breaks the resistance [65]. Thus, it is conceivable that TAV functions as an effector protein suppressing PTI- and ETI-based responses in both Col-0 and Tsu-0 plants: CM1841 TAV is recognized by a specific R protein in Tsu-0, but W260 TAV has evolved its Vir/Avr domain to avoid the recognition. Keeping in line, the W260 TAV, but not TAV from strain D4, could elicit HR in *Nicotiana edwardsonii*, with the TAV N-terminal portion being an Avr determinant [63]. While the strain D4 causes severe systemic infection on *Nicotiana* sp., it produces very mild infection on *A. thaliana* Col-0, unlike the strains W260 or CM1841 [66]. This is despite the fact that transgenic expression of D4 TAV exhibits stronger antisilencing activity in Col-0 than CM1841 TAV [61].Taken together, TAV effector functions in suppression of silencing and PTI/ETI appear to be uncoupled.

Previous studies have implicated SA and ET/JA in regulation of defense against CaMV.However, they produced rather conflicting observations. SA-deficient *A. thaliana* lines NahG, *sid2-2*, *eds5-1*, and *pad4-1* did not show enhanced susceptibility to CaMV strain JI [67,68], suggesting that SA is not required for antiviral defense. On the other hand, mutants *cpr1-1 *and *cpr5-2*, in which SA-dependent defense signaling is activated constitutively, or mutants deficient in ET/JA signaling displayed increased resistance to CaMV [68]. We assume that a potential role of TAV in (partial) suppression of the SA pathway could explain these observations. 

TAV has several interacting host partners that participate in TAV-mediated translation regulation, including TOR (target-of-rapamycin) kinase [69]. Notably, TAV binding activates TOR and TOR-deficient plants are resistant to CaMV [69]. In mammals, TOR is a negative regulator of autophagy and TOR inactivation in response to pathogens promotes innate immune reactions and PCD. As a counter-defense, some viruses including HIV-1 and non-viral pathogens are able to inhibit autophagy to promote infection [70]. In plants, autophagy is required for the development and the innate immune responses to non-viral pathogens. However, it is not clear if the immune responses depend on TOR [70]. It is conceivable that TAV-mediated activation of TOR would inhibit autophagy and thereby suppress innate immune reactions to CaMV. 

Rice tungro disease is caused by synergistic interaction between RTBV and the RNA pricorna-like virus *Rice tungro spherical virus* (RTSV) [71,72]. The tungro-resistant rice cultivars carry genetically separable resistant traits to RTSV and RTBV. A candidate resistant gene for RTSV was recently identified [73], but RTBV-resistance gene is unknown. The RTBV ORF IV of unknown function, which is absent in closely related pararetroviruses, may encode an Avr/effector protein.

## 6. RNA Silencing-Based Antiviral Defense and Viral Silencing Suppressors

RNA silencing is viewed as a primary antiviral defense system of plants [24]. Most plant viruses have RNA genomes and replicate through dsRNA intermediates, which could trigger silencing.Viral replication can potentially be restricted by DCL-mediated processing of the dsRNA intermediates into primary viral siRNAs (vsRNA) and vsRNA-AGO RISC-mediated cleavage of the viral transcripts [74,75]. For some RNA viruses, RDR6 and RDR1 contribute to anti-viral defense by converting viral transcripts to dsRNA precursors of secondary vsRNAs [33,76,77].

Plants infected with DNA viruses of *Caulimoviridae* and *Geminiviridae* accumulate 21, 22 and 24 nt vsRNAs. We and others have shown that all four DCLs (DCL1-4) produce DNA virus-derived vsRNAs [78,79,80,81], whereas only two DCLs (DCL4 and DCL2) produce RNA virus-derived vsRNAs [80,82]. *Arabidopsis dcl1/2/3/4* quadruple mutants, which carry a weak allele of DCL1 (*caf1* or *sin1*) and null alleles of the other DCLs, do not exhibit increased susceptibility to CaMV [81]. This suggests that either defective DCL1 is still able to silence the virus by producing residual vsRNAs, or the innate immune system is still able to partially restrict CaMV infection in the absence of most miRNAs [81]. *dcl1/2/3/4* lines which exhibit even stronger developmental abnormalities than *dcl1* have not yet been tested for susceptibility to non-viral pathogens.

Since DNA viruses do not replicate through dsRNA intermediates, precursors of vsRNAs could potentially be formed by antisense transcription, RDR activity or from secondary structures of viral RNAs. We have established that three functional RDRs (RDR1, RDR2 and RDR6), Pol IV, or Pol V are not required for vsRNA production from CaMV [81] or the geminivirus *Cabbage leaf curl virus* [83] and that vsRNA precursors are likely produced by Pol II-mediated sense and antisense transcription ([83]; reviewed in [75]).

To counteract silencing-based defense, most viruses express suppressor proteins which act at different steps of the silencing process. Viral suppressors have evolved independently as they are structurally diverse and involved in a number of other basic functions in replication, movement and encapsidation. Suppressors of RNA viruses exhibit the following antisilencing activities [74,84]: a) binding long dsRNA and inhibiting DCL4-mediated processing of dsRNA; b) binding siRNA duplexes and interfering with RISC assembly or cell-to-cell movement of siRNAs; c) degrading siRNAs; d) targeting AGO1 and possibly other AGOs for degradation; e) binding AGO1 and inactivating AGO1-RISC; f) inducing miR168 to block AGO1 translation from miR168-targeted AGO1 mRNA. As noted above, targeting AGO1 by viral suppressors can also have an impact on innate immunity.

Interestingly, none of the above antisilencing strategies was reported for DNA viruses. In geminiviruses, AC2, AC4, V2 and betaC1 exhibit suppressor activities. AC2, the most studied geminiviral suppressor, is a transcriptional activator with its nuclear localization required for suppression, likely through transcriptional activation of endogenous silencing suppressors [85]. Additionally, AC2-mediated inactivation of cytoplasmic components of the methyl cycle leads to suppression of DNA methylation in the nucleus, which may promote transcription of viral DNA [75,86]. AC4 binds single-stranded sRNA and cooperates with AC2 to synergistically enhance disease symptoms, possibly by suppressing a cytoplasmic step of silencing [87]. V2 interacts with SGS3, a co-factor of RDR6 [88] and can outcompete SGS3 in binding an asymmetric RNA duplex having a long 5’-overhang *in vitro* [89], suggesting that it may interfere RDR6-dependent production of viral siRNAs. On the other hand, V2 also binds CYP1, a member of the family of papain-like cysteine proteases which are involved in plant defense against diverse pathogens [90]. The geminiviral betasatellite DNA encodes the pathogenicity factor betaC1 which has been implicated in suppression of silencing [91] and JA responses [92,93], providing a link to innate immunity. Consistent with the latter finding, JA treatment of *Arabidopsis thaliana* plants disrupts geminivirus infection [94]. It remains to be investigated whether geminiviral silencing suppressors are capable of suppressing PTI and/or ETI. 

In pararetroviruses, so far only CaMV TAV has been reported as a silencing suppressor. Our work [61] and other studies [59,60] suggest that TAV interferes with processing of RDR6-dependent dsRNA by DCL4, which would block secondary siRNA production and silencing amplification. TAV does not exert its suppressor activity through binding to long dsRNA (our unpublished data) and does not suppress silencing induced by RDR6-independent dsRNA [61]. TAV interacts with the dsRNA-binding protein DRB4, a partner of DCL4 [60]. However, DRB4 and other members of the DRB family, except for the DCL1 partner DRB1 [80], are not required for the biogenesis of CaMV vsRNAs and *drb* mutants do not exhibit increased susceptibility to virus infection (unpublished data). Thus, TAV interaction with DRB4 may be unrelated to suppression of antiviral silencing. As discussed above, the interactions of a viral effector (such as TAV) with critical host proteins could be monitored by the innate immune system and lead to immune responses. Intriguingly, nuclear localization of TAV is required for silencing suppression: mutation in the TAV nuclear localization signal (but not its nuclear export signal) interfered with TAV ability to block accumulation of tasiRNAs [60].

CaMV infection and TAV expression do not alter AGO1 protein levels [81]. Furthermore, TAV does not affect the cleavage of tasiRNA precursors by AGO1-miR173, AGO2-siRNA [95], or AGO7-miR390 [96] RISCs. All these AGOs contribute to defense against RNA viruses, and AGO1 and AGO2 were shown to bind vsRNAs [76,97,98]. Thus, CaMV must evade vsRNA-AGO RISC-mediated silencing by a different mechanism. 

Some pararetrovirus genera including *Tungrovirus* and *Badnavirus *do not possess a TAV homolog [56], suggesting that other viral protein(s) may have effector activity. Alternatively, these viruses employ a different strategy to evade silencing and innate immune responses. Recently, we have uncovered a CaMV strategy of silencing evasion based on viral RNA, which can potentially operate in all genera of plant pararetroviruses. pgRNA of most pararetroviruses has a long leader sequence preceding the first large viral ORF; this leader folds into a stable stem-loop structure bypassed by the ribosome during the initiation step of pgRNA translation [58,99,100,101]. Our surprising finding was that, in CaMV-infected *A. thaliana*, the 600 bp leader region spawns massive quantities of 21-, 22-, and 24-nt vsRNAs, comparable to the entire complement of host siRNAs and miRNAs, while other regions of the 8 kb CaMV genome spawn little vsRNAs [81]. This finding and other lines of experimental evidence [81] indicate that massive production of leader vsRNAs does not restrict viral gene expression but serves as a decoy to divert the silencing machinery from the promoter and protein-coding regions of the CaMV genome [81]. It is also conceivable that this massive siRNA production may have an impact on PTI-based innate responses regulated by RNA silencing. 

The dsRNA precursor of CaMV leader-derived vsRNAs is composed of 8S RNA, which was previously identified in CaMV-infected turnip to cover the entire leader, and its antisense copy. Since genetic evidence excludes involvement of RDR1, RDR2, RDR6, Pol IV or Pol V in production of 8S dsRNA and vsRNAs [81], 8S dsRNA is likely produced by Pol II in the nucleus. CaMV replicates in the cytoplasm via reverse transcription of pgRNA (35S RNA). CaMV virions contain open-circular dsDNA with a gap in the minus strand marking the start of reverse transcription primed by Met-tRNA and two gaps in the plus strand marking initiation sites of plus strand DNA synthesis. At each replication cycle, virions release viral DNA into the nucleus where the gaps are repaired and the resulting covalently-closed dsDNA is transcribed by Pol II [56,57]. The 35S transcript covers the whole genome with a 200 nt terminal repeat. The subgenomic 19S RNA (mRNA for TAV) is transcribed from a separate promoter but shares 3´ terminus with 35S RNA. *Ca.* 600 nt 8S RNA has the same 5´ terminus as 35S RNA and ends near the gap in the minus strand DNA, suggesting that it is produced owing to abrupt termination of Pol II transcription on a fraction of viral DNA having the unrepaired gap. We assume that 8S RNA folds into viroid-like secondary structure that can be converted by Pol II into dsRNA. Indeed, plant RNA viroids are replicated by Pol II through dsRNA intermediates [102]. Our findings imply that a large and stable stem-loop structure of the pgRNA leader in pararetroviruses has evolved to be both a good substrate for Pol II that generates a dsRNA decoy engaging all DCLs in massive production of vsRNAs, and a poor target for AGO-RISC charged by vsRNAs of antisense polarity. Likewise, viroid is a good source of siRNAs but itself is resistant to RISC due to inaccessibility of its highly structured RNA [103]. TAV gene is a later acquisition in evolution of pararetroviruses, which enables a more efficient mechanism of pgRNA translation [58] and ensures that limited, vsRNA-directed cleavage of pgRNA and other ways of aberrant RNA production do not trigger RDR6-dependent amplification secondary vsRNAs.

Moissiard and Voinnet [79] reported that CaMV leader-derived vsRNAs have the potential to target host transcripts. Our studies revealed that portions of the CaMV leader are dispensable and can be functionally replaced by distinct primary sequences from RTBV [101,104,105], which is not consistent with a deliberate strategy of virus-induced silencing of host transcripts that would rely on sequence-specific recognition of target mRNAs. Targeting host transcripts may be collateral damage of the massive vsRNA production.

It remains to be established if a CaMV-like decoy strategy is used by RTBV and other plant pararetroviruses and perhaps animal pararetro- and retroviruses with structured leaders [58]. Interestingly, human adenovirus expresses short, highly-structured RNAs that suppress the interferon-mediated antiviral defense and sequester Dicer [106]. Thus, an RNA decoy strategy to counteract host defenses has evolved in both plant and animal viruses.

## 7. Conclusions

In this review we have described growing evidence that innate immunity (PTI and ETI) and RNA silencing function in concert to defend plants against viruses and other pathogens. To counteract this dual defense and establish infection in susceptible hosts, pathogens have evolved effectors with a primary function to suppress both PTI/ETI innate responses and RNA silencing. In resistant host plants, effectors (also called Avr proteins) are recognized by plant NB-LRR proteins, which results in ETI/PCD-based resistance. DNA pararetroviruses have evolved two types of effectors, a viral protein-based and a viral RNA-based. The pararetrovirus CaMV-encoded effector/Avr protein TAV interferes with RDR6-dependent secondary siRNA production likely required for amplification of antiviral silencing and, according to indirect evidence, suppresses PTI/ETI-based innate responses. Likewise, the pararetrovirus RTBV ORF IV of unknown function may suppress silencing and/or innate immunity. In CaMV, the pgRNA leader region spawns massive amounts of vsRNAs and the precursor of these vsRNAs, 8S dsRNA, serves as a decoy engaging critical components of the silencing machinery. The mechanisms of biogenesis and action of CaMV 8S dsRNA and its presumptive counterpart in RTBV and other pararetroviruses remain to be further investigated.
